# Emerging and Reemerging Parasitic Diseases in Taiwan: A Retrospective Study of Clinical Case Reports in 2001~2018

**DOI:** 10.3390/pathogens13050383

**Published:** 2024-05-05

**Authors:** Shao-Lun Hsu, Chia-Kwung Fan

**Affiliations:** 1 Department of Molecular Parasitology and Tropical Diseases, School of Medicine, College of Medicine, Taipei Medical University, Taipei 11031, Taiwan; arinahsu@gmail.com; 2 Dr. Rolf M. Schwiete Center for Limbal Stem Cell and Congenital Aniridia Research, Saarland University, 66421 Homburg, Germany; 3 Department of Ophthalmology, Saarland University Medical Center, 66421 Homburg, Germany; 4 Research Center of International Tropical Medicine, College of Medicine, Taipei Medical University, Taipei 11031, Taiwan; 5 Cell Physiology and Molecular Image Research Center, Wan Fang Hospital, Taipei Medical University, No. 111, Section 3, Xinglong Road, Wen-Shan District, Taipei 11696, Taiwan

**Keywords:** parasites, retrospective study, neglected tropical diseases (NTDs), Taiwan, global health

## Abstract

Emerging and re-emerging parasitic diseases can cause significant economic burdens at national and global levels. However, governments often underestimate or ignore these diseases, especially in developed countries. This retrospective, case-oriented study analyzed parasitic diseases reported in Taiwan between 2001 and 2018. One hundred and thirty-two eligible clinical profiles of Taiwanese patients obtained from the NCBI, Scopus, Google Scholar, and Web of Science databases and local journals according to age, sex, source of infection, symptoms, risk factors, and geographical regions were analyzed. The analysis results showed that the number/frequency of cases caused by nematodes (46.97%) or protozoa (37.88%) was significantly higher than that of trematodes (9.85%) or cestodes (5.30%) (*p* < 0.0001). Northern Taiwan (46.97%) had a significantly higher rate than Southern Taiwan (33.33%), Central Taiwan (8.33%), and Eastern Taiwan (5.30%) (*p* < 0.05). The 15–65 age group (68.94%) had a significantly higher rate than the 65–90 age group (22.73%) and the 0–15 age group (8.33%) (*p* < 0.0001). Males (70.46%) had a significantly higher number/frequency of cases than females (29.54%) (*p* < 0.0001). People who acquired the infection through the food/soil route (32.58%) or who had a low immune status (32.58%) had a higher rate than travel-related infections (15.15%) (*p* < 0.001). The present study showed that emerging/reemerging parasitic infections continue to be of great concern to the lives and health of Taiwanese citizens and, if ignored, will threaten the health of the Taiwanese people; therefore, the delineation of preventive measures by health authorities is urgently warranted.

## 1. Introduction

Neglected tropical diseases (NTDs) belong to a heterogeneous group of communicable diseases that are prevalent in tropical and subtropical areas in over 149 countries, and they predominately affect the rural poor and urban slum dwellers who have inadequate sanitation, low educational levels, and close contact with domestic animals or infectious vectors [1,2,3,4]. Approximately 40 diseases fall under the category of NTDs, including schistosomiasis, dengue fever, soil-transmitted helminthiases (STHs), and many other pathogens from a wide range of viruses, bacteria, fungi, and parasites [1]. More than 1.4 billion people suffer one or more NTDs worldwide (including more than 500 million children), and NTDs cost billions of dollars each year in developing economies and pose significant threats to at-risk populations by entrapping them in a prolonged state of poor health and nutrition [2,3]. It was suggested by the World Health Organization (WHO) in 2007 at the first meeting of global partners that most NTDs could be effectively controlled or even in many cases possibly be eradicated by 2020 [5]. 

In Taiwan, in the past few decades, parasitic diseases have become rare because of extensive control measures, including massive screening for intestinal parasitic infections among schoolchildren, along with the implementation of public health education and improved hygienic conditions [6,7,8]. However, rapid globalization has thus resulted in the emergence and reemergence of various parasitic infections in Taiwan, either due to indigenous spread or imported via immigration [9,10]. Recent studies showed incidence rates of intestinal parasitic infections among laborers from Southeast Asian countries were pretty high as assessed through the routine physical examinations required for the approval of work permits each year [11,12]. Along with improvements in cross-strait relations within the past 20 years, growing numbers of Taiwanese tourists are traveling to endemic areas such as Southeast Asia, China, and Africa, and globalization trends have also allowed the rapid transmission of parasitic diseases from international students, immigrants, and migrant workers [13]. From another point of view, growing populations suffer from systemic diseases and pathogens like the human immunodeficient virus (HIV). Still, the use of immunosuppressants in clinics nowadays contributes to an immunodeficiency state of patients, which consequently contributes to the emerging relevance of parasitic infections in Taiwan [14,15]. 

Although parasitic diseases continuously cause impacts on public health in Taiwan that cannot be ignored and thus must be better understood and addressed, they are primarily neglected and overshadowed by HIV, tuberculosis (TB), and cancers, which have come to the forefront of public focus in Taiwan’s society owing to rapid modernization and globalization [16]. To facilitate an understanding of the status and patterns of emerging and reemerging parasitic diseases in Taiwan, this study assesses 127 case reports of parasitic infections in Taiwan in 2001–2018. It summarizes the key clinical features of each case. We further discuss the trends, challenges, and impacts of various factors influencing the prevalence and management of parasitic diseases in Taiwan. As the first study attempts to shed light on clinical profiles of various parasitic infections in Taiwan on a large scale, the present study can provide simplified data to clinical workers for both diagnostic and preventive strategies in the future more efficiently.

## 2. Materials and Methods 

### 2.1. Search Strategy

We designed a case-oriented approach by searching the literature released online and published in local journals in both English and Chinese. Four English databases (PubMed, Scopus, Google Scholar, and Web of Science) and one Chinese database (Airiti Library, Taiwan) were searched for published papers from January 2001 to December 2018. The current review was carried out using medical subject heading (MeSH) terms including: “Taiwan”, “scientific name of parasite” (list in Table 1), and “case”. Using a few keywords alone or combined to search for and eliminate some unqualified papers by manual assessments, we tried to include all eligible papers with minimal personal errors.

### 2.2. Study Selection and Data Extraction

**Figure 1:**
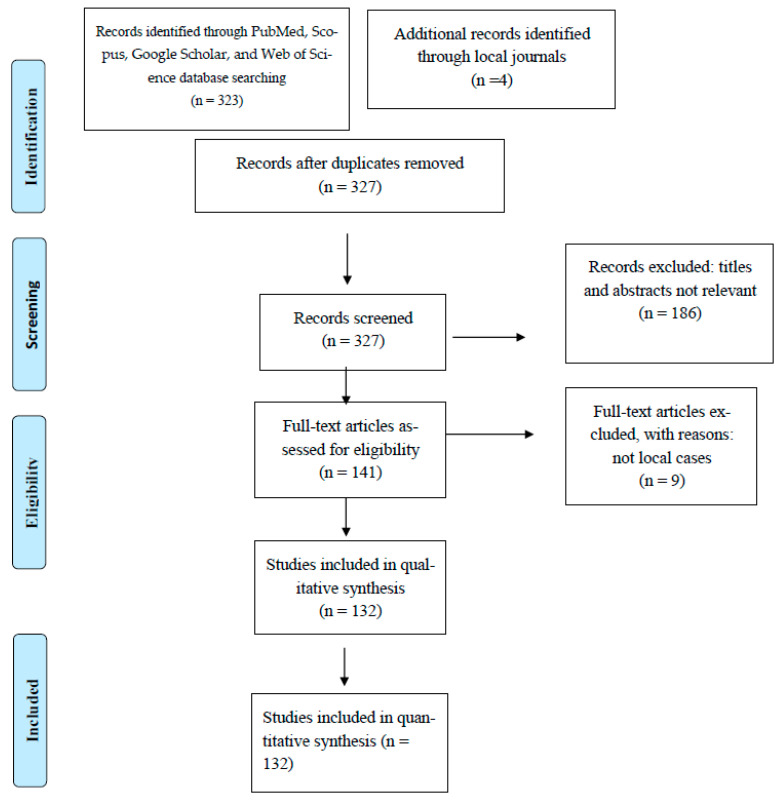
Study selection and data extraction were used from PubMed, Scopus, Google Scholar, Web of Science databases, and local journals, and the candidate articles from 2001 to 2018 were selected according to the inclusion criteria.

According to the inclusion criteria, case reports of parasitic infections among Taiwanese nationals published in 2001–2018 were included. To assess the eligibility criteria, all papers were reviewed by three independent reviewers. Epidemiological investigations, including mass surveys and screening in aboriginal tribes, elementary schools, or endemic areas, were excluded due to their incomplete information on follow-up analyses and consequences, which would have caused deviations due to the relatively large amount of data added. In addition, foreigners with parasite infections before entering Taiwan who were accidentally detected in Taiwan were also excluded from the present study. However, cases from foreigners who acquired parasite infection during their residential period in Taiwan and Taiwanese nationals who were infected by the parasite after traveling abroad or engaging in short-term exchanges were regarded as indigenous and imported cases, respectively, in the present study (Figure 1). 

### 2.3. Descriptive Analysis

The desired data were precisely gathered using a data extraction form based on gender, age, geographic region, and the classification of infectious agents. Etiologies corresponding to each parasitic infection were referenced to the Centers for Disease Control and Prevention (CDC) criteria. The item of ‘city’ defined for reported cases was the county/city of the locality of the hospital rather than the location where the infection was detected or emerged. Clinical risk factors and symptoms presented in clinics were also excerpted from the original data and classified into subcategories by keywords. 

**Figure 2:**
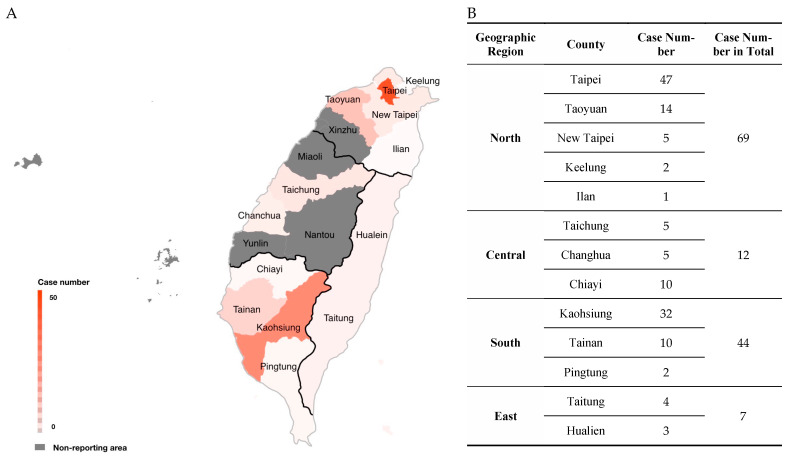
(**A**) Distribution and (**B**) the number of clinical parasitic cases by searching the literature released online and published in local journals in both English and Chinese in different geographical areas along with different cities/counties in Taiwan from 2001 to 2018.

Figure 2 shows the frequency distributions of infectious cases by different parasitic species and geographic areas. To avoid bias, we evaluated the distribution tendency according to geographic regions instead of counties since most of the cases reported in the northern region were from Taipei City. In contrast, most reported cases in the southern region were from Kaohsiung City. A histogram was used to indicate relationships between infectious cases and age groups, which were divided into child (<15 years old), adult (15~65 years old), and elderly groups (≥65 years old) according to the definition of the National Development Council (NDC).

### 2.4. Statistical Analysis

Differences in the prevalence of nematode, trematode, cestode, and protozoan infections, as well as regions and routes of infection, and univariate crude odds ratio (OR) with 95% confidence interval (CI) to determine associations between independent variables, risk factors, and infection were calculated using SAS v.9.3 software (SAS Institute, Cary, NC, USA), and a *p*-value of <0.05 was considered statistically significant.

## 3. Results

Of the 327 case reports retrieved from the databases, 132 eligible cases of parasitic infection in 2001–2018 in Taiwan were recruited and are summarized in Table 1. Results of the literature search of each study, including patient gender and age, geographic region, parasite species, etiology, risk factors, symptoms, and characteristics, were further analyzed.

### 3.1. Common Parasite Species in Clinics

A total of 132 case reports met the criteria for inclusion in this study. The number of reported cases in the parasite category from highest to lowest was 62 for nematodes, 50 for protozoa, 13 for trematodes, and 7 for cestodes. The number/frequency of cases caused by nematodes (46.97%, 62/132) was significantly higher than that by trematodes (9.85%, 13/132) or by cestodes (5.30%, 7/132) (OR = 8.11, 95% CI= 4.16–15.79, *p* < 0.0001; OR = 15.82, 95%CI = 6.87–36.44, *p* < 0.0001).

It is noteworthy that *Angiostrongylus cantonensis* and *Strongyloides stercoralis* were the top parasitic infections by nematodes, and comprised 13 and 11 cases, respectively. Compared to *Ang. cantonensis* or *S. stercoralis* infections, a total of 14 hookworm infections (6 cases of *Ancylostoma duodenale*, 3 cases of *A. ceylanicum*, 2 cases of *Necator americanus,* and 3 unknown hookworm infections) could be considered as equally significant. All eight cases of *Trichinella papuae* infection came from an outbreak in 2008 after the consumption of contaminated soft-shelled turtles sold at a local Japanese restaurant (Table 1). 

Seventeen cases of infection with *Acanthamoeba* spp. were recorded, directly related to improper cleaning of contact lenses or orthokeratology lenses, particularly in the young and middle-aged groups. As for other protozoan infections, *Toxoplasma gondii* and *Entamoeba histolytica* infections were highly associated with raw food consumption and a low immunity state, including immune deficiency syndrome and patients with long-term use of immunosuppressants (Table 1).

### 3.2. Geographic Distributions of Parasite Infections

Most reported cases of parasite infections in Taiwan were from the northern and southern regions, with total numbers of 69 and 44 cases, respectively, in 2001~2018. In contrast, there were only 19 cases reported in the rest of Taiwan, including 12 from central Taiwan and 7 from eastern Taiwan. The regional infection rate was significantly higher in northern Taiwan (52.27%, 69/132) than in southern Taiwan (33.33%, 44/132), central Taiwan (8.33%, 11/132), and eastern Taiwan (Figure 2A,B) (OR = 2.19, 95%CI = 1.33–3.60, *p* = 0.002; OR = 12.05, 95%CI = 5.95–24.39, *p* < 0.0001; OR = 19.56, 95%CI = 8.49–45.05, *p* < 0.0001). 

### 3.3. Age and Gender Distributions of Parasite Infections

The number/frequency of cases was significantly higher in the age group of 15–65 yrs (68.94%, 91/132) than in the age group of 65–90 yrs (22.73%, 30/132) or the 0–15 yrs age group (8.33%, 11/132) (OR = 7.55, 95%CI = 4.36–13.07, *p* < 0.0001; OR = 24.42, 95%CI = 11.90–50.11, *p* < 0.0001) (Table 2). Regarding the gender difference, males (70.46%, 36/132) had a significantly higher number/frequency of cases than females (29.54%, 14/132) (OR = 3.16, 95%CI = 1.61–6.20, *p* < 0.001) (Table 2).

### 3.4. Characteristics of High-Risk Populations

The risk factors associated with different parasite infections vary according to their life cycle. In the present study, significant categories, e.g., food/soil-borne (32.58%, 43/132), low immune status (32.58%, 43/132), and travel history (15.15%, 20/132), are important factors contributing to parasitic infections in Taiwan (Table 3). The infection rate was significantly higher for food/soil-borne infections than for travel-related infections (OR = 2.71, 95%CI = 1.49–4.93, *p* < 0.001) (Table 3), whereas it did not show any difference compared to a low immune status (Table 3).

Farmers have a risk factor of walking barefoot in their fields, which is highly associated with soil-transmitted nematode infections, e.g., *Anc. duodenale*, *Anc. ceylanicum,* and *N. americanus*; consumption of raw foods, including sashimi, lettuce, and even frogs, field snails, and snake blood, used in traditional Chinese medicine are common sources of infection, resulting in high incidence rates of *Ang. cantonensis* and sporadic cases of *Anisakis simplex* infections. A striking example is the strong positive association between improper contact lens washing and *Acanthamoeba* spp. infections. The risk factors mentioned above, i.e., being a farmer, having an immunodeficiency status, and international population movements, all contribute to the dominant tendency for parasitic infections, especially nematode and protozoan infections that predominantly occur in the male working-age population in Taiwan (Table 2).

### 3.5. Clinical Symptoms of Parasitic Infections

Parasitic diseases commonly present with mild and non-specific symptoms in the clinic (Table 4). Our results indicate that a slight fever, abdominal discomfort, and generalized malaise were most commonly described in our 132 clinical reports (Table 4). Abdominal discomfort, including intermittent pain, diarrhea, and abdominal distention, is related to most food-borne parasites and may lead to weight loss and malnutrition in patients. Clinical symptoms probably correspond to the lifecycle of the different parasites, as infections involving the respiratory system generally present with dyspepsia and coughing. At the same time, red eyes and stromal infiltration are often observed with ocular infections (Table 4). 

## 4. Discussion

This case-oriented study summarized 132 clinical profiles of parasitic diseases in Taiwan from 2001 to 2018. We attempted to provide simplified data for clinical workers to have a comprehensive understanding of these NTDs and to present important information on emerging/reemerging parasitic diseases that, if ignored, will threaten the health of Taiwanese; therefore, health authorities should delineate preventive measures. The weighted total number of parasitic infections in Taiwan was obtained using descriptive statistics rather than data mining because parasitic infections are often sporadic in developed countries and excessive analysis could lead to spurious cause-and-effect relationships. The results demonstrated high-risk factors and common symptoms of parasitic diseases, which were per our prior knowledge. 

It should be noted that this study had some limitations, such as (I) a lack of invariable cut-off values to determine common risk factors and symptoms, (II) some symptoms were not detected in the clinic or risk factors may have been inadvertently omitted by patients, (III) a lack of studies in some regions of the country, and (IV) large-scale epidemiological surveys, infections among foreigners, and other unpublished cases from our laboratory (Department of Molecular Parasitology and Tropical Disease, School of Medicine, Taipei Medical University) were excluded because of their incomplete information. These limitations may have introduced bias and should be improved in future work. Nevertheless, we believe that relative comparisons are still statistically meaningful based on the same method of data selection and keyword extraction used in this study.

To the best of our knowledge, this is the first case-oriented study and analysis to provide a general overview of parasitic diseases in Taiwan and the first study to reveal the emergence of migration trends and low immune status as two potential risk factors that may play an important role in parasitic infections not only in Taiwan but also in other developed countries today [17,18,19,20]. 

Our results showed that 11.36% of parasitic infections were imported cases, of which 41.69% and 35.28% came from Southeast Asian countries and China, respectively. Epidemiological studies showed a high possibility of disease transmission between Southeastern China and Taiwan [17]. If cross-strait relations (between Taiwan and China) continue to improve and the tendency to travel abroad increases, international travelers introducing parasitic diseases from nearby areas such as China, Southeast Asia (together, these two accounted for 84.9% of imported cases) and even Africa (imported malaria) will become more common. 

There are many factors behind the increasing prevalence of parasitic diseases. The influx of migrant workers began in 1989 when the government’s policy to address the labor shortage in Taiwan allowed foreign workers to work on 14 construction projects [21]. Commercial interactions and immigration trends between Taiwan and Southeast Asian countries have contributed to disease transmission mostly via the fecal–oral route, e.g., *Ascaris* and *Trichuris* [21,22]. Many studies have documented a disproportionately higher endemicity of parasitic diseases among migrant workers and other non-citizen residents [10,22], which is consistent with our hypothesis. Cheng et al. described an infection rate of 37.7% for *Blastocystis hominis* and other intestinal parasites among Vietnamese migrant women who married Taiwanese men in southern Taiwan [10]. They also showed an increasing trend in infections throughout observation. Wang also documented this phenomenon among migrant workers, finding an average infection rate of 8.2% among foreign workers from the Philippines, Thailand, and Indonesia [23]. Notably, women in this study had a much higher infection rate (11.7%). This higher infection rate among women was further confirmed by Hsieh et al. [24]. We propose that an integrated preventive control program be adopted and continued as a regular protocol for immigrants and Taiwanese citizens who like to travel abroad. Both of these populations are subject to routine health examinations for intestinal parasite screenings upon entering or re-entering Taiwan. However, only foreign students applying for visas require parasite screening by stool examination to complete their Health Certificate (Form B) for General Resident Visa Applicants. Results of studies by Hsieh et al. reported an infection rate among foreign students of 4.6% [13]. This is lower than other populations entering Taiwan but higher than local infection rates [6]. The migration of international populations exacerbates the indigenous spread of parasitic diseases [23]. Similar situations have been reported by Monge-Maillo et al. [25], who attempted to determine the most common infectious diseases among two mobile immigrant groups of sub-Saharan Africans and Latin Americans in Spain and found that the most common diagnoses were latent tuberculosis (32.6%), filariasis (19.2%), chronic hepatotropic viral infections (19.2%), intestinal parasites (11.0%), and malaria (9.6%), as well as by Laukamp et al. [26] who examined the disease spectrum of unaccompanied asylum-seeking adolescents (UASAs) from sub-Saharan Africa in Germany and found that the two most common infections were helminths and *Giardia intestinalis* with a high prevalence of 33.2%, representing a high cost to the public health system and possible transmission within the host country. Although cross-strait relations (between Taiwan and China) have been somewhat strained recently, bilateral trade is intense, and more than 500,000 Taiwanese businesspeople now work in China, where the prevalence of helminth infection is estimated at 21.74% [27]. In addition, more than 2 million Taiwanese have traveled to China in the last 20 years, but no studies have been conducted to determine whether this population is at risk of introducing indigenous parasites from China to Taiwan, which should be of serious concern [28]. 

As noted above, higher prevalence rates of parasitic infections have been observed in populations with hypoimmunity [24]. Our results showed that several reasons could lead to a low immune status in patients, including immunodeficiency syndrome, use of immunosuppressants, or undergoing systemic disease or chemotherapy. We also found that people with HIV infection and organ transplant recipients were the highest-risk groups. One reported case in Taiwan was of a patient with end-stage renal disease who acquired *Plasmodium vivax* infection after kidney transplantation. The kidney was from an Indian donor, which means that organ transplantation is not only associated with the use of immunosuppressants after surgery but also with the possibility of organs from questionable sources [29]. This risk was highlighted in a study by Robert-Gangneux et al., who reported 87 cases of Toxoplasma infection in European transplant recipients between 2010 and 2014 [30]. Despite the low prevalence of parasitic diseases in Europe, this study confirmed that toxoplasmosis is still a clinical problem in transplant recipients. Furthermore, Abanyie et al. also indicated that from the CDC experience in 2009–2013, 11 out of 20 (55%) solid organ recipients appeared symptomatic due to *S. stercoralis* infection and noted that two of them died from complications of strongyloidiasis [31]. In addition to organ transplantation, HIV-induced hypoimmunity plays a critical role in opportunistic infections (OIs). OIs are a major cause of morbidity and mortality in immunocompromised individuals due to low cellular and humoral immune function [32,33]. A report by Nissapatorn et al. indicated infections such as *Cryptosporidium parvum*, *Isospora belli*, *Leishmania tropica*, *Tox. Gondii,* and *S. stercoralis* are the top five parasite-related diseases in the OI category [34]. According to studies by Nissapatorn et al. [34] and Lee et al. [35], HIV infection became a notifiable disease in Taiwan in 1984, and 33,423 patients had been confirmed by the end of 2016. After 30 years of the HIV epidemic, parasitic infections have become one of the most common AIDS-related OIs (AOIs) in both developed and developing countries [36,37]. Although the annual number of reported HIV infections continues to increase, few studies have investigated the epidemiology of parasitic diseases, such as *E. histolytica*, in HIV-infected patients in Taiwan [38,39]. The Taiwanese health authorities have unfortunately neglected the importance of parasitic diseases in Taiwan by not funding national surveys on the status of parasitic infections in Taiwan for almost 30 years. In comparison, China regularly monitors parasitic disease trends every 10 years [40,41]. Due to the long neglect of parasitic infections by the government, more and more doctors and technicians are unfamiliar with parasitic diseases. In addition, parasitic infections are now overshadowed by other diseases such as HIV, TB, and cancer, which has led to disproportionate funding for research. All these factors have created a policy environment that may lead to the resurgence of parasitic diseases in Taiwan. Altogether, the role of water, food, tourism, and demographic changes all contributed to the spread of these diseases that cannot be ignored.

The present study has shown that parasitic infections remain highly relevant to the lives and health of Taiwanese citizens. They potentially threaten the affected population by trapping them in a prolonged state of poor health and nutritional deprivation, reducing quality-adjusted life years, and further limiting economic and social improvement opportunities. Our findings suggest that appropriate intensive screening of at-risk populations and long-term commitment to monitoring infectious trends in Taiwan should be policy and funding priorities, but it is also crucial that the medical community, including general practitioners and other medical doctors, is alerted and educated about these diseases. They should be equipped to identify the needs and gaps in the country’s healthcare system. In conclusion, understanding the clinical profiles of current parasitic diseases, the larger political and migration contexts, and the hypoimmunity syndrome currently present in Taiwan is paramount for effectively treating and preventing emerging/reemerging parasitic diseases. Health authorities must reprioritize understanding and managing these important public health issues.

## Figures and Tables

**Table 1:** One hundred and thirty-two Taiwanese patients infected with different parasite species were searched in PubMed, Scopus, Google Scholar, Web of Science, and a Chinese database (Airiti Library, Taiwan) from 2001 to 2018.

Name	Parasite Species	Case Number
Nematodes (*N* = 62)		
	*Ancylostoma ceylanium*	3
	*Ancylostoma duodenale*	6
	*Angiostrongylus cantonensis*	13
	*Anisakis simplex*	1
	*Ascaris lumbricoides*	1
	*Capillaria philippinensis*	3
	*Dirofilaria immitis*	3
	*Enterobius vermicularis*	2
	Hookworm (unknown)	3
	Filaria	1
	*Necator americanus*	3
	*Strongyloides stercoralis*	11
	*Thelazia callipaeda*	1
	*Trichinella papuae*	8
	*Trichuriasis trichiura*	3
Trematodes (*N* = 13)		
	*Clonorchis sinensis*	4
	*Fasciola hepatica*	1
	*Paragonimus westermani*	4
	*Schistosoma japonicum*	4
Cestodes (*N* = 7)		
	*Diphyllobothrium latum*	3
	*Diphyllobothrium monsoni*	2
	*Taenia asiatica*	1
	*Taenia solium*	1
Protozoa (*N* = 50)		
	*Acanthamoeba castellanii*	1
	*Acanthamoeba spp.*	17
	*Blastocystis hominis*	1
	*Cryptosporidium hominis*	1
	*Entamoeba histolytica*	8
	*Leishmania tropica*	4
	*Naegleria fowleri*	1
	*Plasmodium falciparum*	3
	*Plasmodium ovale*	1
	*Plasmodium vivax*	2
	*Toxoplasma gondii*	9
	*Trichomonas vaginalis*	2

**Table 2:** The number of clinical parasitic cases was distributed differently across different age groups and genders in Taiwan from 2001 to 2018 by searching the literature released online and published in local journals in both English and Chinese.

Age (Years)	Trematodes	Cestodes	Nematodes	Protozoa	Total
0~15	0	2	2	7	11
15~65	6	5	42	38	91
65~90	7	0	18	5	30
gender (M:F)	9:4	2:5	46:16	36:14	93:39

**Table 3:** The number of clinical parasitic cases distributed in different high-risk populations in Taiwan from 2001 to 2018 by searching the literature released online and published in local journals in both English and Chinese.

Category	Risk Factor	No. of Cases	Category	Risk Factor	No. of Cases
Food/soil-borne infections (*N* = 43)	raw fish ingestion	10	Systematic disease/low immune status (*N* = 43)	diabetes mellitus	6
	raw seafood ingestion (soft-shelled turtle)	8		systematic disease/underlying disease	16
	raw vegetable ingestion	7		organ transplant recipient/immunodeficiency syndrome	16
	drinking uncooked stream water/contaminated water contact	5	*Acanthamoeba* spp. infection	wearing contact lenses	10
	raw snail ingestion/contact with snails	3		wearing orthokeratology lenses	5
	raw frog ingestion/used frog skin as a wound dressing	4	Identity and career	aboriginal people	8
	raw meat (food) consumption	6		farmers	19
Travel-related infections (*N* = 20)	worked/lived/traveled in China	8	-	homosexual man/oral–vaginal sexual relations	3
	worked/lived/traveled in Southeast Asia	9	-	contact with dogs	3
	worked/lived/traveled in Africa	3	-	others	19

**Table 4:** Distribution and number of parasitic cases across different clinical symptoms in Taiwan from 2001 to 2018 by searching the literature released online and published in local journals in both English and Chinese.

Category	Symptoms	No. of Cases	Category	Symptoms	No. of Cases
Non-specific symptoms	headaches	11	Ocular involvement	visual disturbances	8
	fever	36		foreign-body sensation in the eye/painful eye	9
	chills	7		red eye	7
	nausea	11		eye stromal infiltration/ground-glass edema	7
	vomiting	14	CNS system involvement	mental deterioration	8
	poor appetite	10		paresthesias	8
	general malaise/fatigue	24		nuchal rigidity	6
	limb weakness	6	Respiratory tract involvement	coughing	7
	myalgias	7		dyspnea	8
	edema	12	Skin involvement	erythematous plaques or nodules	7
*GI tract involvement	abdominal pain	29	Others	-	50
	abdominal distention	8			
	diarrhea/borborygmus	26			
	excreted worms during defecation	5			
	melena/stool occult blood	17			
	weight loss	11			

*GI, gastrointestinal.

## Data Availability

Not applicable.
